# In Vitro Antimicrobial and Immunomodulatory Activity of Trans-4-Hydroxycinnamic Acid Against Bovine Mastitis-Associated Bacteria

**DOI:** 10.3390/antibiotics15101008

**Published:** 2026-10-10

**Authors:** Shaguneet Kour, Neelesh Sharma, Natesan Balaji, Sandeep Kour, Ankit Magotra, Young-Ok Son

**Affiliations:** 1 Division of Veterinary Medicine, Faculty of Veterinary Science & Animal Husbandry, Sher-e-Kashmir University of Agricultural Sciences & Technology of Jammu, R.S. Pura, Jammu 181102, Jammu and Kashmir, India; shaguneetk@yahoo.com (S.K.); balanate248@gmail.com (N.B.); drsandeepkour87@gmail.com (S.K.); 2 Division of Animal Genetics & Breeding, Faculty of Veterinary Science & Animal Husbandry, Sher-e-Kashmir University of Agricultural Sciences & Technology of Jammu, R.S. Pura, Jammu 181102, Jammu and Kashmir, India; ankitoms@gmail.com; 3 Department of Animal Biotechnology, Faculty of Biotechnology, College of Applied Life Sciences and Interdisciplinary Graduate Program in Advanced Convergence Technology and Science, Jeju National University, Jeju 690756, Republic of Korea

**Keywords:** mastitis, AMR, trans-4-hydroxycinnamic acid, qRT-PCR

## Abstract

**Background/Objectives:** Bovine mastitis is a major economic and health challenge in the dairy industry, heavily dependent on antibiotics and increasingly complicated by antimicrobial resistance (AMR). This study evaluated the in vitro therapeutic efficacy of trans-4-hydroxycinnamic acid (4-HCA) as a novel alternative therapeutic agent on bovine mammary epithelial cells (bMECs). Specifically, the objectives were to assess the biofilm-forming capacity of mastitis-associated bacterial isolates, determine the antibacterial and bactericidal profile of 4-HCA, and investigate its immunomodulatory effects on infected bMECs using quantitative real-time PCR (qRT-PCR). **Methods:** A panel of fourteen bovine mastitis-origin bacterial isolates was evaluated for biofilm production using both the tube method and the microtiter plate assay. The antibacterial activity of 4-HCA was determined via agar well diffusion, disc diffusion, minimum inhibitory concentration (MIC), and minimum bactericidal concentration (MBC) assays. Cytotoxicity and safe working concentrations of 4-HCA on bMECs were established using the MTT cell-viability assay (3.125 to 800 µg/mL). Furthermore, an in vitro inflammatory model using bMECs infected separately with *Staphylococcus aureus* and *Escherichia coli* (1 × 10^6^ CFU/mL) was treated with 4-HCA (50 and 100 µg/mL) or ciprofloxacin. Relative transcriptional expression profiles of pro-inflammatory cytokines (IL-1, IL-6, and TNF- α) were quantified via qRT-PCR and analyzed using the comparative 2^−ΔΔCT^ method. **Results:** Quantitative biofilm evaluations identified 19% to 22% of the bacterial isolates as strong biofilm producers and 25% to 31% as moderate producers, with prominent biofilm formation observed in *Enterococcus faecalis, Raoultella planticola, Shigella sonnei*, and *Staphylococcus aureus.* 4-HCA exhibited robust bactericidal efficacy with mean MIC values ranging from 0.625 to 1.25 mg/mL and MBC values from 2.5 to 10 mg/mL. The MTT assay confirmed 100% bMEC viability at the experimental treatment concentrations of 50 and 100 µg/mL. qRT-PCR analyses revealed that 4-HCA treatment significantly modulated (*p* < 0.05) pro-inflammatory cytokine transcript profiles. In the S. aureus model, treatment significantly downregulated TNF-alpha expression while differentially upregulating IL-1 and IL-6 mRNA levels, demonstrating cytokine-specific immunomodulation. Conversely, in the E. coli model, 4-HCA induced a concentration-dependent reduction across all evaluated pro-inflammatory cytokines (IL-1, IL-6, and TNF- α). **Conclusions:** Trans-4-hydroxycinnamic acid demonstrates significant in vitro antibacterial activity, biofilm disruption potential, and targeted immunomodulatory effects on bovine mammary epithelial cells. These findings establish a strong foundational basis for exploring 4-HCA as a promising alternative therapeutic agent for managing bovine mastitis and mitigating antimicrobial resistance in veterinary medicine.

## 1. Introduction

Etymologically derived from the Greek terms mastos, meaning breast or udder, and the suffix -itis, denoting inflammation, mastitis is clinically defined as an inflammatory condition of the mammary gland [1]. It remains one of the most prevalent and economically consequential disorders affecting dairy livestock [2,3,4]. As a multi-etiological pathology intrinsically linked to production systems and housing environments, mastitis manifests through characteristic physical, chemical, and bacteriological alterations in mammary parenchyma [5]. Pathologically, the condition is driven by the host’s innate immunological defense mechanisms reacting to localized noxious stimuli, predominantly microbial colonization and proliferation within the mammary gland [6].

Bovine mastitis imposes a substantial financial burden on the Indian dairy economy, with total annual losses estimated at approximately INR 7165.51 crores, of which subclinical mastitis accounts for 57.93% (INR 4151.16 crores) [7]. Complementary economic analyses indicate that national losses average approximately INR 1390 per lactation per cow, predominantly driven by reduced milk yield (49%) and direct veterinary intervention costs (37%) [8].

Antibiotics are currently the primary treatment of choice for bovine mastitis [9]. Nevertheless, their effectiveness is declining due to antibiotic resistance, which is regarded as a major worldwide health issue [10]. Therefore, in order to achieve the best possible health for humans, animals, and the environment, we must increase awareness of the importance of including a multisectoral One Health strategy when addressing complex issues like AMR [11].

Antimicrobial resistance (AMR) represents a critical global health crisis and an escalating focus of scientific inquiry, marked by a 45% expansion in research output between 1999 and 2018 [12]. Notwithstanding this heightened academic engagement, substantial knowledge gaps persist, notably concerning longitudinal AMR trajectories across international, regional, and national scales, as well as distinct operational sectors including public health, veterinary medicine, and agricultural production. These critical deficiencies continue to impede the formulation and implementation of evidence-based policy frameworks.

Therefore, it was essential to formulate alternative and more effective control and therapeutic strategies, such as vaccines, bacteriophage therapy, herbal medicine, homeopathy, etc., especially to treat resistant forms of bacteria in bovine mastitis cases. One of the many alternatives tested was the successful use of phytochemicals. Phytochemicals are bioactive compounds present in medicinal plants. Phytochemical constituents, including flavonoids (e.g., quercetin, kaempferol, catechins), alkaloids (e.g., berberine, quinine, morphine), terpenoids and terpenes (monoterpenes, sesquiterpenes, triterpenes), phenolic acids (e.g., gallic acid, caffeic acid, ferulic acid), saponins, tannins, and essential oil derivatives (e.g., thymol, carvacrol, eugenol, linalool), exhibit broad-spectrum antimicrobial efficacy. These bioactive compounds exert their bactericidal and bacteriostatic effects through diverse cellular targets, encompassing the inhibition of cell wall biosynthesis, permeabilization of cytoplasmic membranes, suppression of nucleic acid replication, disruption of intermediary metabolic pathways, and interference with bacterial cell-to-cell signaling mechanisms (quorum sensing) [10].

The structural development of bacterial biofilms commences with the initial adhesion of planktonic cells to abiotic surfaces, driven primarily by electrostatic or hydrophobic forces. This preliminary phase is subsequently consolidated by surface-associated adhesins, including flagella, pili, and fimbriae, which establish stable structural bridges anchoring the microbial cells to the substratum. Following attachment, the bacteria proliferate to construct a complex, multicellular community consolidated by a matrix of extracellular polysaccharides. This structure eventually achieves a dynamic equilibrium upon reaching a critical biomass, triggering the detachment of the outermost cell layers. These liberated planktonic cells can then migrate and colonize novel surfaces [13].

In *Staphylococcus aureus*, this process is prominently supported by the secretion of an exopolysaccharide matrix known as the glycocalyx. This matrix mediates adherence to host cells, including mammary epithelial cells, and optimizes nutrient acquisition. Concurrently, it grants the pathogen the resilience to endure high-shear physiological environments and survive external stressors, effectively shielding it from host phagocytosis, disinfectants, and antimicrobial agents [14]. This protective barrier represents a primary mechanism of recalcitrance to conventional antibiotic therapies; the matrix impedes drug penetration, restricting the localized antibiotic exposure to sub-inhibitory concentrations (sub-MIC) that fail to achieve pathogen eradication. Consequently, there is an urgent need for alternative therapeutic strategies that specifically target and disrupt the biofilm-forming mechanisms of *S. aureus* [15].

Trans-4-hydroxycinnamic acid (4-HCA) also known as Trans-p-Coumaric Acid, C_9_H_8_O_3_, is a notable phenolic compound classified within the hydroxycinnamic acid family. Within veterinary pharmacology and immunology, it is receiving increasing attention for its capacity to modulate host immune responses and attenuate systemic inflammation. The anti-inflammatory mechanisms of 4-HCA are mediated through the suppression of pro-inflammatory cytokines such as TNF-α, IL-1β, and IL-6; the downregulation of key inflammatory enzymes including cyclooxygenase-2 (COX-2) and inducible nitric oxide synthase (iNOS); and the inhibition of mitogen-activated protein kinase (MAPK) phosphorylation. Furthermore, 4-HCA exerts immunomodulatory effects by directing macrophage polarization—promoting the transition from the classically activated pro-inflammatory (M1) phenotype to the alternatively activated tissue-repair (M2) phenotype- alongside regulating T-cell-mediated responses [16]. 4-HCA exhibits profound antimicrobial and antibiotic-sensitizing efficacy against multidrug-resistant (MDR) pathogens, by disrupting bacterial membrane potential, promoting cytoplasmic acidification, and enhancing antibiotic uptake mechanism [17]. It plays an increasingly critical role as resistance-modifying agent to combat rising antimicrobial recalcitrance, although plant-derived phenylpropanoids typically exhibit moderate intrinsic antibacterial capabilities. When integrated into combinatorial regimens, 4-HCA acts synergistically with conventional antibacterial classes, significantly enhancing the efficacy of drugs like chloramphenicol and amoxicillin, particularly at low antibiotic dosages. This cooperative action is primarily driven by targeted mechanisms involving cell membrane disruption and the inhibition of critical bacterial virulence factors, offering an effective strategy to lower individual drug requirements and mitigate broader antimicrobial resistance mechanisms [18].

## 2. Results

### 2.1. Bacterial Isolates

The list of bovine mastitis-origin bacterial isolates used for this research work are mentioned in Table 6.

### 2.2. Estimation of Biofilm Formation

#### 2.2.1. Tube Method

**Figure 1:**
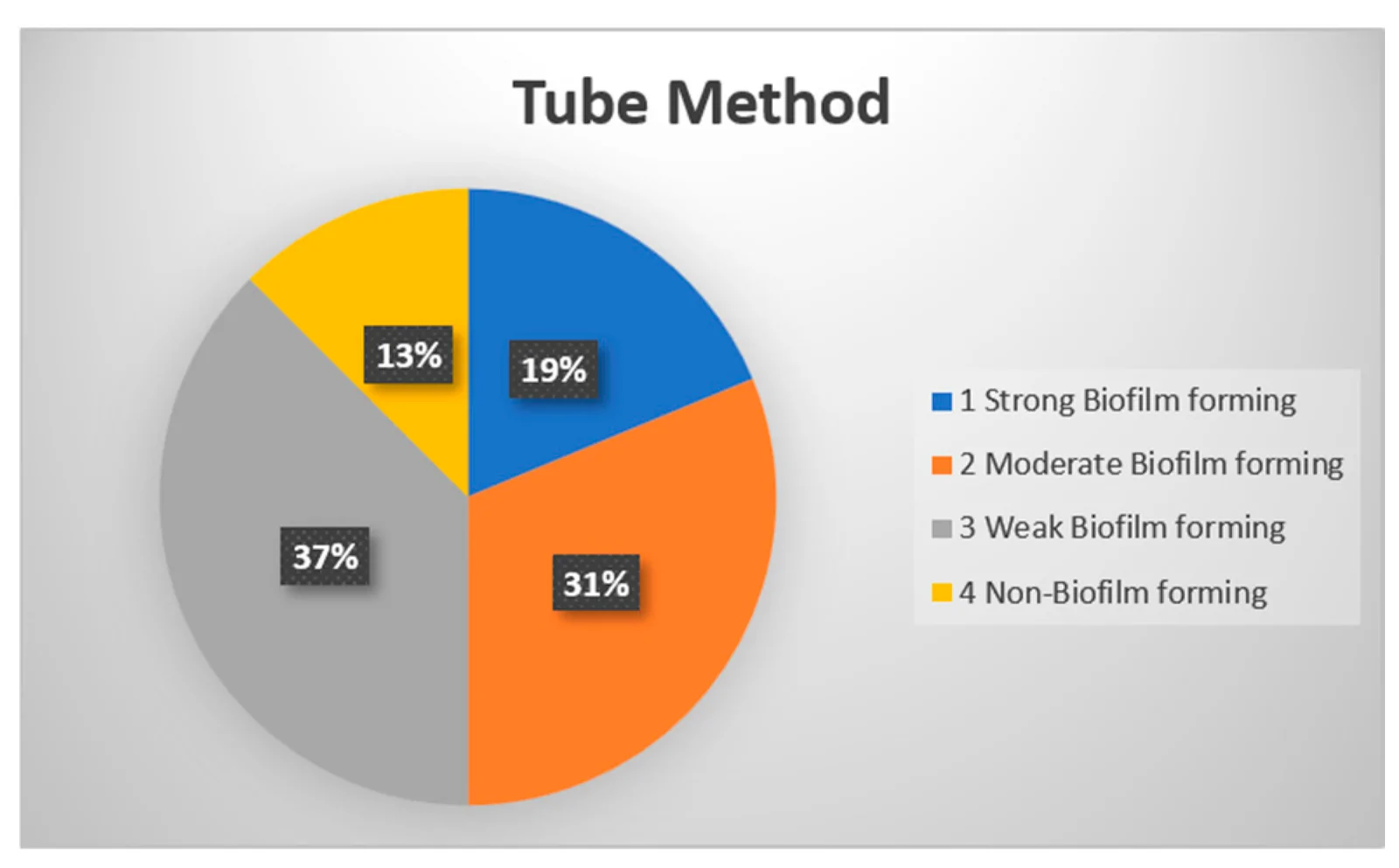
Quantitative distribution of biofilm-forming phenotypes determined by the Tube Method.

**Figure 2:**
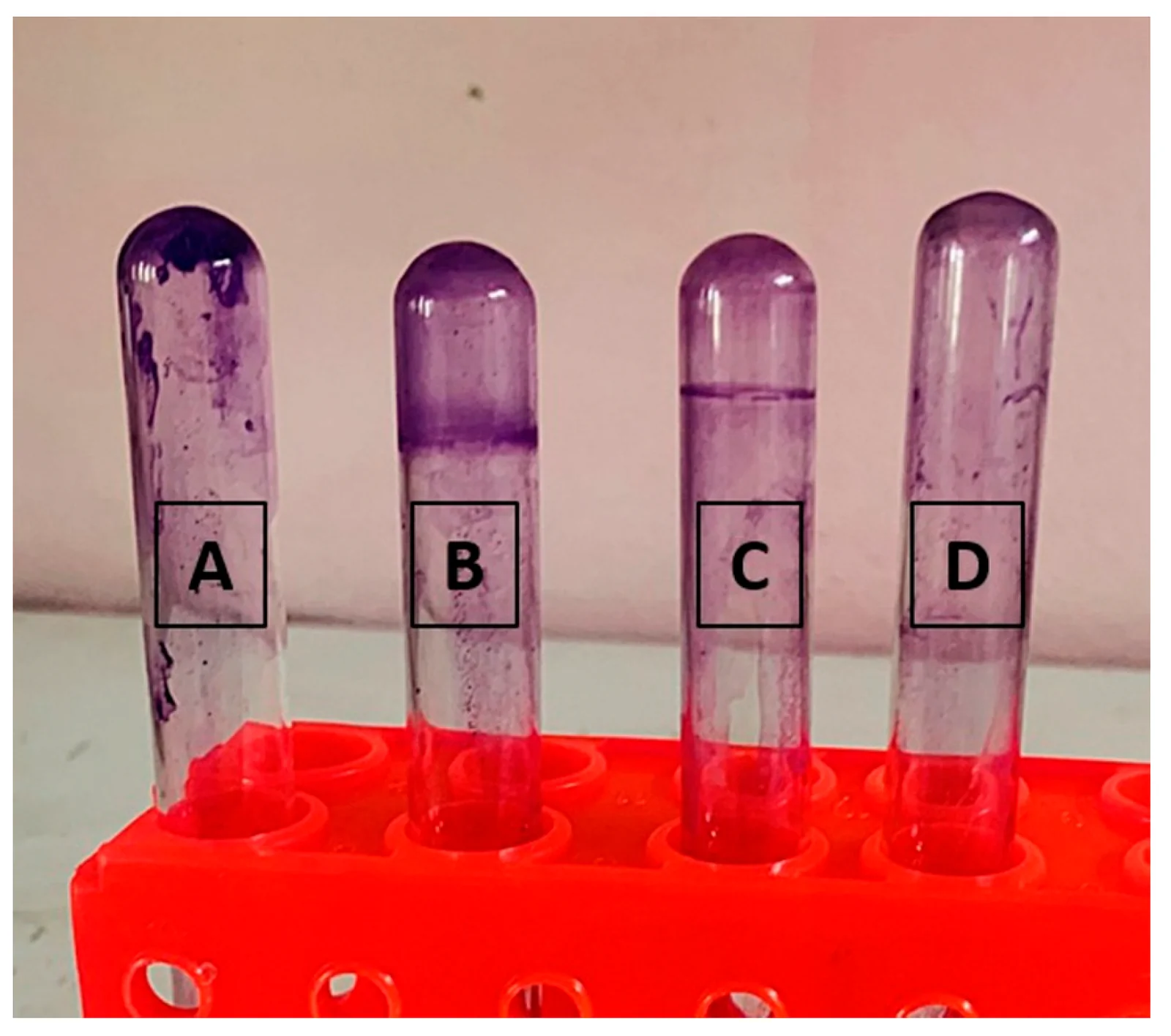
Categorization of bacterial isolates based on quantitative biofilm formation capacity: (A) strong, (B) moderate, (C) weak, and (D) non-biofilm producers.

Quantitative biofilm evaluation revealed that 31% of the tested isolates functioned as moderate biofilm producers, whereas 19% exhibited strong biofilm formation, 37% demonstrated weak biofilm capabilities, and 13% were classified as non-biofilm producers (Figure 1). Pronounced biofilm development was predominantly observed in *Enterococcus faecalis*, *Raoultella planticola*, *Shigella sonnei*, and *Staphylococcus aureus*. Moderate biofilm production was documented across *Pseudomonas putida*, *Staphylococcus epidermidis*, *Staphylococcus aureus*, and *Enterococcus faecium*. Conversely, weak biofilm phenotypes were detected in *Serratia liquefaciens*, *Streptococcus agalactiae*, and *Proteus mirabilis*, while *Escherichia coli* strains were identified as non-biofilm producers (Figure 2).

#### 2.2.2. Microtiter Plate Assay

**Figure 3:**
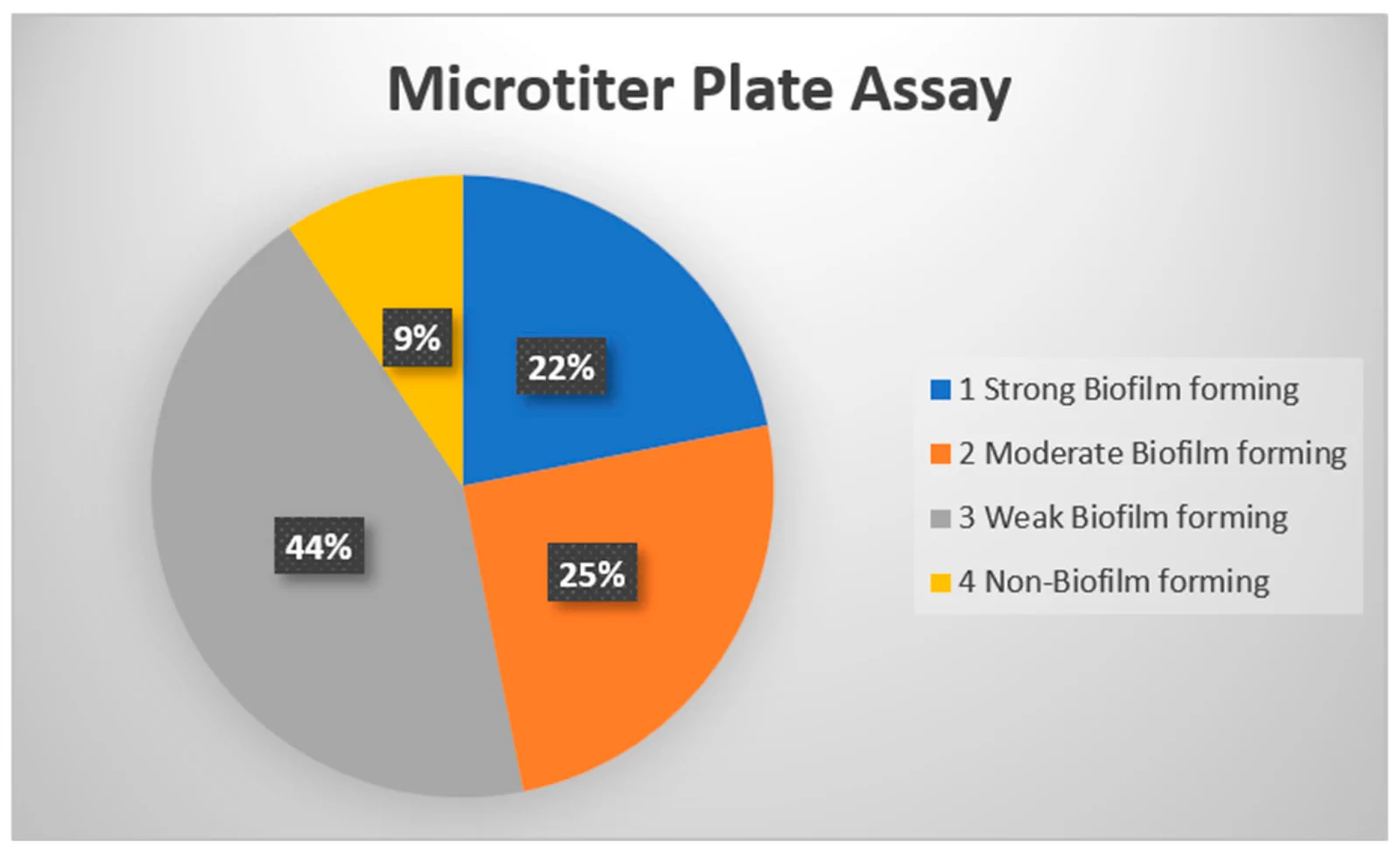
Quantitative distribution of biofilm-forming phenotypes determined by the Microtiter Plate Assay.

**Scheme 1:**
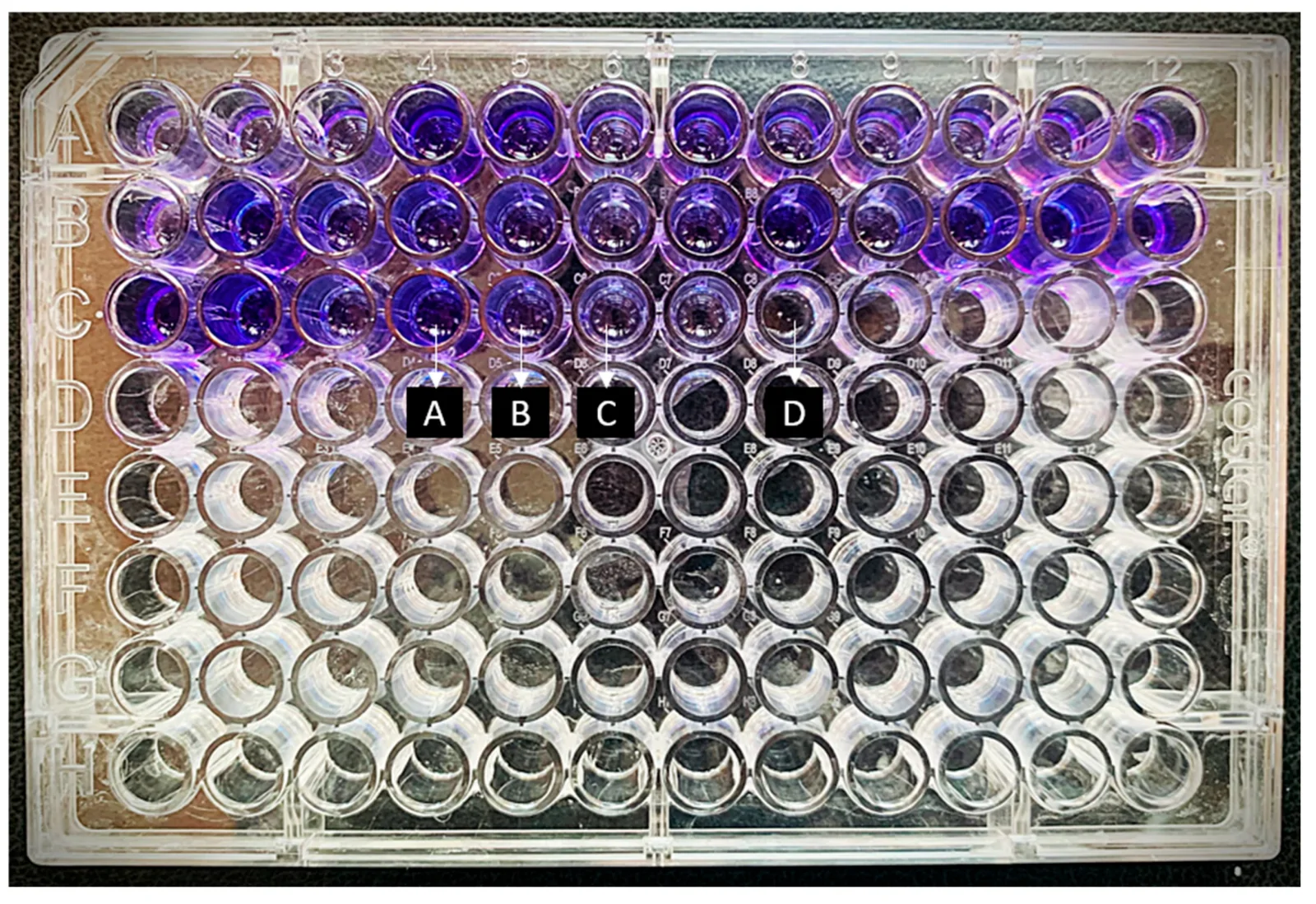
Categorization of bacterial isolates based on quantitative biofilm formation capacity: (A) strong, (B) moderate, (C) weak, and (D) non-biofilm producers.

Biofilm quantification demonstrated that 25% of the isolates functioned as moderate producers, 22% exhibited strong biofilm development, 44% displayed weak biofilm capabilities, and 9% were classified as non-biofilm producers (Figure 3). Prominent biofilm formation was identified in *Enterococcus faecalis*, *Hafnia alvei*, and *Klebsiella quasipneumoniae*. Moderate biofilm production was documented among *Pseudomonas putida*, *Staphylococcus epidermidis*, *Staphylococcus aureus*, and *Enterococcus faecium*. Weak biofilm phenotypes were recorded in *Serratia liquefaciens*, *Streptococcus agalactiae*, and *Proteus mirabilis*, whereas *Escherichia coli* isolates were characterized as non-biofilm producers (Scheme 1).

### 2.3. Antimicrobial Activity of Phytochemical

#### 2.3.1. Agar Well Diffusion Method

**Figure 4:**
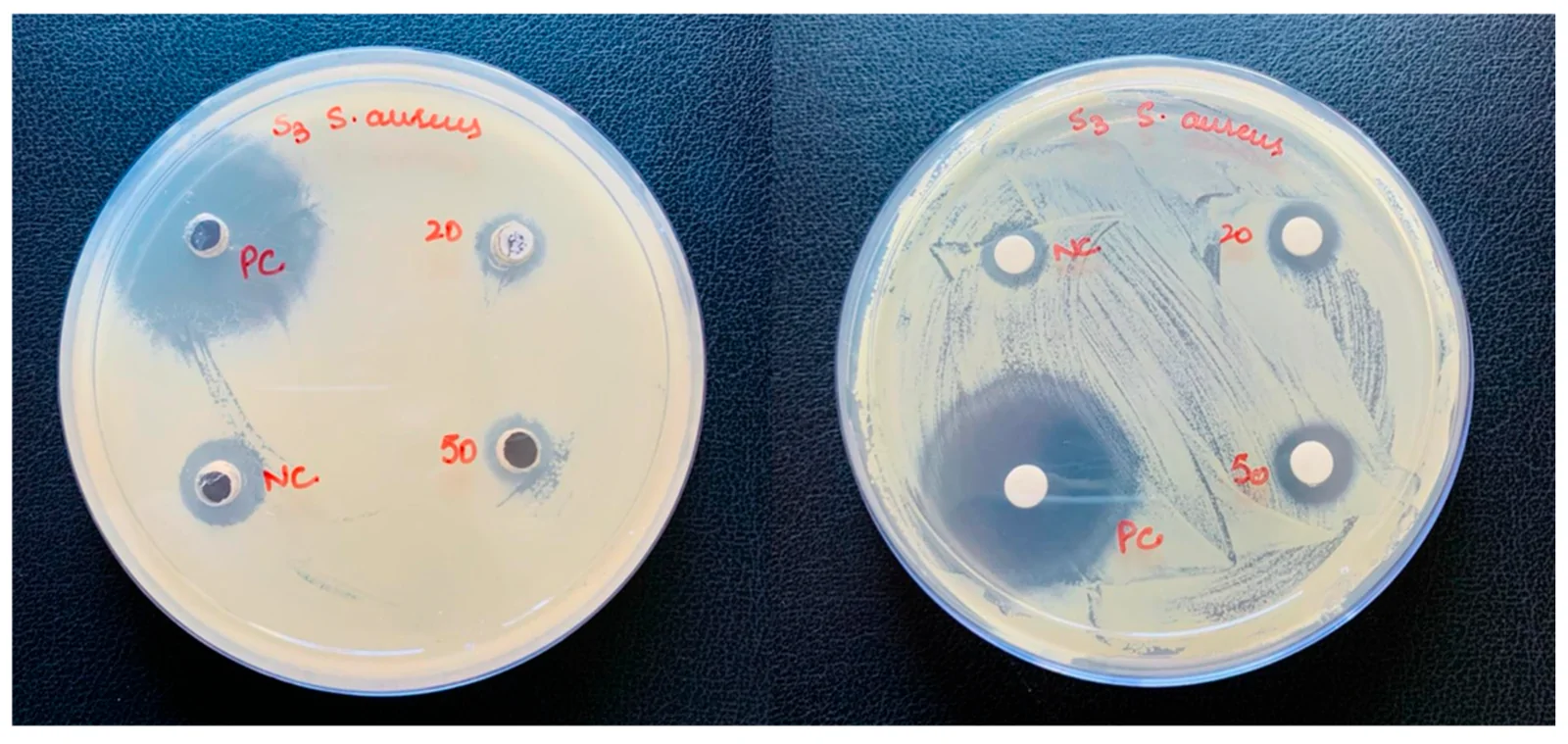
Antimicrobial activity of 4-HCA at a concentration of 20 mg/ml and 50 mg/ml against *S. aureus*. Each well was loaded with 50 μL of phytochemical.

It was revealed that the maximum zone of inhibition shown was 10 mm against isolates like *S. aureus*, *E. faecalis*, *Pseudomonas putida*, *Shigella sonnei*, *Streptococcus agalactiae*, *Klebsiella quasipneumoniae*, and *Serratia liquefaciens. Raoultella planticola*, *Proteus mirabilis*, and *Hafnia alvei* showed partial zones of inhibition (Figure 4).

#### 2.3.2. Disc Diffusion Method

It was revealed that the maximum zone of inhibition of 12 mm was formed against *S. aureus* at 20 mg/ml and 50 mg/mL concentrations. Zones of inhibition of 11 mm and 12 mm at 20 mg/mL and 50 mg/mL concentrations, respectively, were shown by *P. mirabilis*. Zone of inhibition of 10 mm was shown by *E. faecium*, *Pseudomonas putida, S. epidermis, Shigella sonnei,* and *Serratia liquefaciens. Raoultella planticola* again showed a partial zone of inhibition similar to the disc diffusion method. *Streptococcus agalactiae* and *Hafnia alvei* showed no zone of inhibition (Figure 4). The reference drug, ciprofloxacin, produced a standard zone of inhibition of 20 mm.

#### 2.3.3. Minimum Inhibitory Concentration (MIC) and Minimum Bactericidal Concentration (MBC)

A total of 14 bacterial isolates were evaluated in this assay. For minimum inhibitory concentration determinations, the initial test well concentration was established at 10 mg/mL. The compound 4-HCA demonstrated measurable in vitro inhibitory activity, with MIC values ranging from 0.625 to 1.25 mg/mL and MBC values ranging from 2.5 to 10 mg/mL across the evaluate (Table 1).

#### 2.3.4. Quantitative Real-Time Polymerase Chain Reaction (qRT-PCR)

An in vitro experiment for assessing the cytokine-modulatory activity of 4-HCA was performed using two mastitis models, i.e., *S. aureus*-induced inflammatory model and *E. coli*-induced inflammatory model (Table 2).

##### 2.3.4.1. *S. aureus*-Induced Inflammatory Model

**Figure 5:**
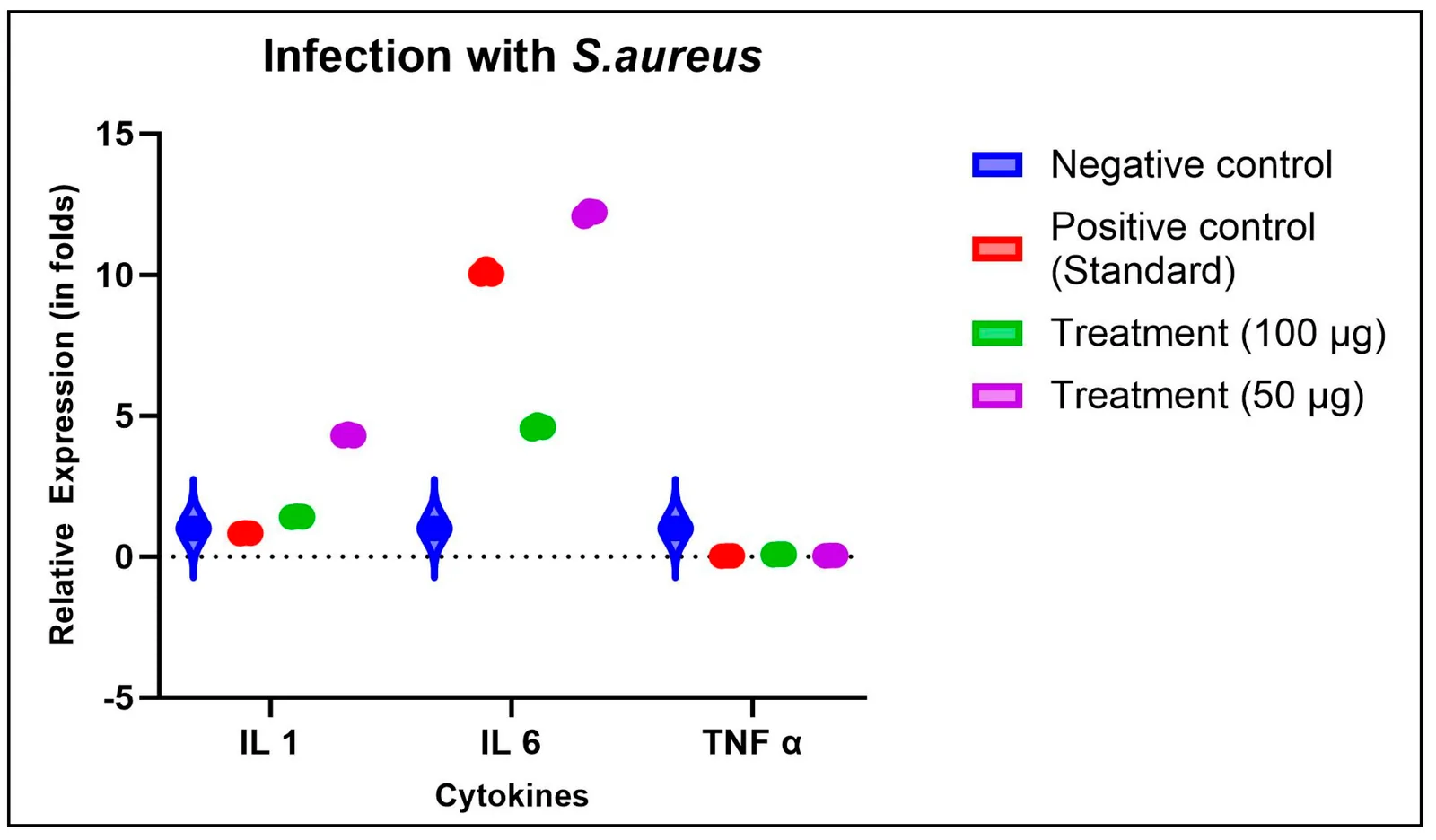
Relative expression of IL-1, IL-6, and TNF-α in *S. aureus*-infected bMECs. Statistical differences among all four experimental groups were evaluated using two-way ANOVA followed by Tukey’s multiple-comparison test. Values are presented as mean ± SEM.

**Figure 6:**
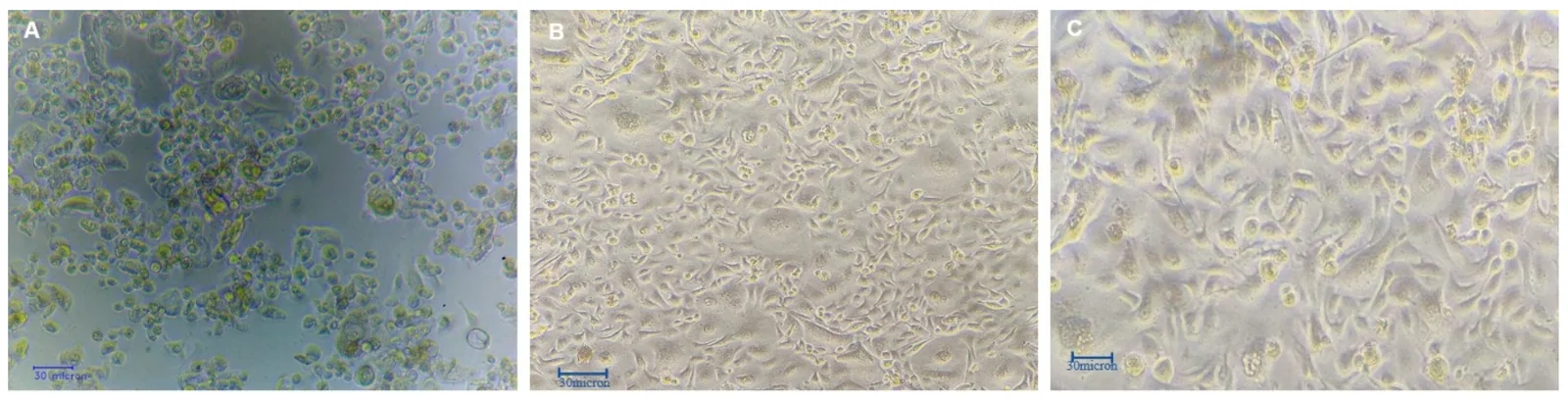
Image representing growth and morphology of bMECs (negative control) in basal growth medium, after 6 h of infection with *S. aureus* (1 × 10^6^ CFU/mL). (**A**) bMECs showed necrotic changes and vacuolation under a contrast inverted microscope (10×). (**B**) Very few bMECs showed necrotic changes after treatment under a contrast inverted microscope (10×). (**C**) Very few bMECs showed necrotic changes after treatment under a contrast inverted microscope (10×).

The relative gene expression ratio of the qPCR results of *S. aureus*-infected groups shows that there is a 0.96–0.97-fold downregulation of treatment group against the TNF-α gene and 0.42–3.35-fold upregulation in the treatment group against IL-1 and 3.6–11.2-fold upregulation in the treatment group against IL-6 (Table 2; Figure 5). This means the suggested phytochemical reduced the expression of TNF-α, which is a proinflammatory cytokine that is involved in proliferation, differentiation and activation of many immune cells such as B lymphocytes and NK (natural killer) cells along with the stimulation and release of other cytokines. In contrast to TNF-α, IL-1 and IL-6 expression increased in the 4-HCA-treated groups. Therefore, the response cannot be interpreted as a uniform anti-inflammatory effect and is more appropriately described as cytokine-specific immunomodulation [19]. Morphological changes in bMECs were observed after treatment in all groups, with different degrees of necrosis (Figure 6).

##### 2.3.4.2. *E. coli*-Induced Inflammatory Model

**Figure 7:**
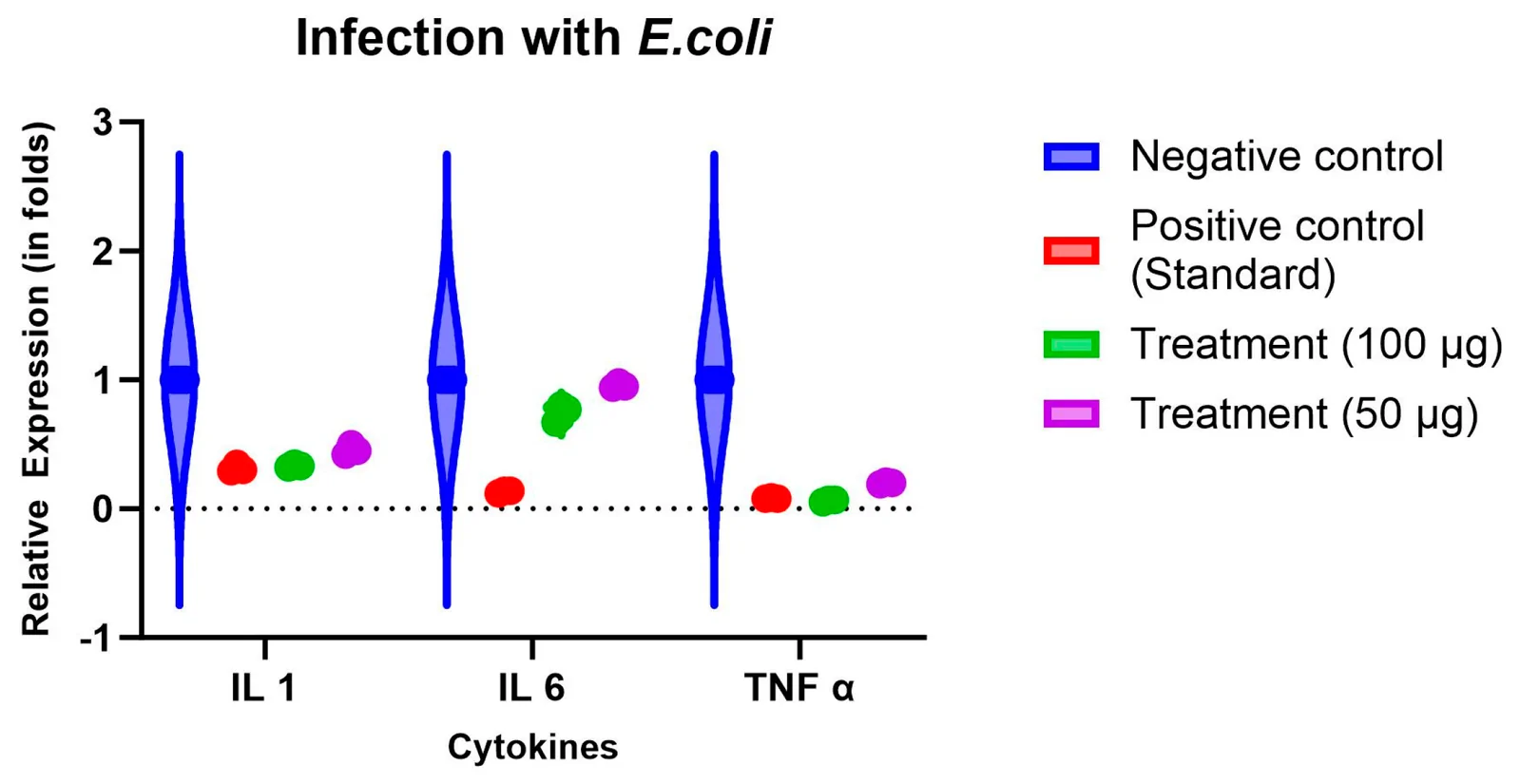
Relative expression of IL-1, IL-6, and TNF-α in *E. coli*-infected bMECs. Statistical differences among all four experimental groups were evaluated using two-way ANOVA followed by Tukey’s multiple-comparison test. Values are presented as mean ± SEM.

**Figure 8:**
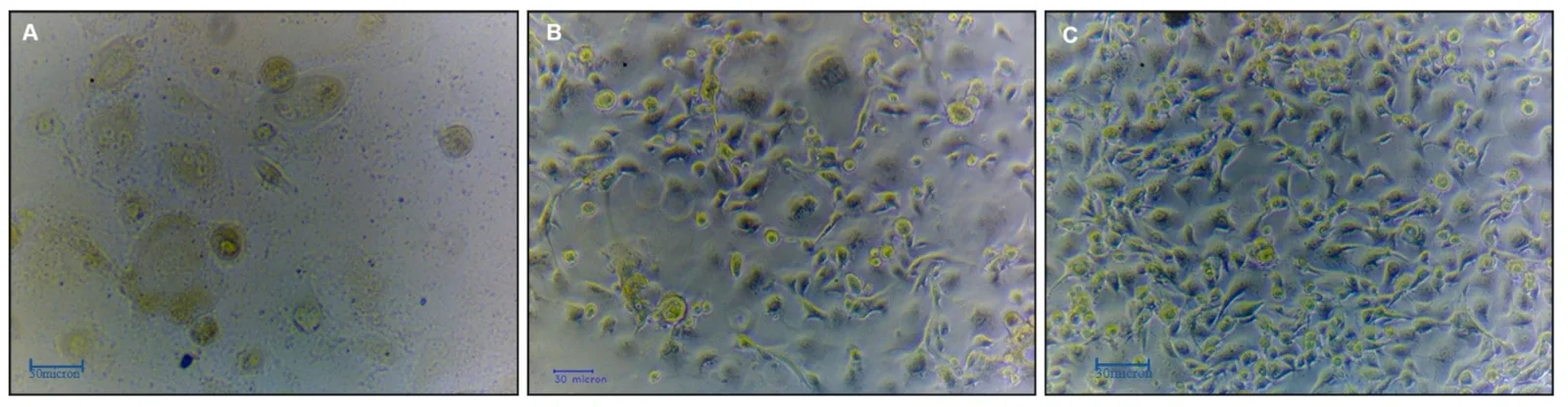
Image representing growth and morphology of bMECs (negative control) in basal growth medium, after 6 h of infection with *E. coli* (1 × 10^6^ CFU/mL). (**A**) bMECs showed necrotic changes and vacuolation under a contrast inverted microscope (10×). (**B**) Very few bMECs showed necrotic changes after treatment under a contrast inverted microscope (10×). (**C**) Very few bMECs showed necrotic changes after treatment under a contrast inverted microscope (10×).

In the *E. coli*-infected bMEC model, 4-HCA treatment reduced the expression of all three evaluated cytokines. IL-1 expression decreased to 0.33-fold and 0.46-fold following treatment with 100 and 50 μg/mL 4-HCA, respectively. IL-6 expression decreased to 0.75-fold and 0.96-fold, whereas TNF-α expression decreased to 0.06-fold and 0.20-fold at 100 and 50 μg/mL, respectively. These findings indicate a concentration-dependent reduction in pro-inflammatory cytokine expression in the *E. coli*-infected bMEC model (Table 3; Figure 7). Morphological changes in bMECs were observed after treatment in all groups, with different degrees of necrosis (Figure 8).

### 2.4. Effect of 4-HCA on bMEC Viability

**Figure 9:**
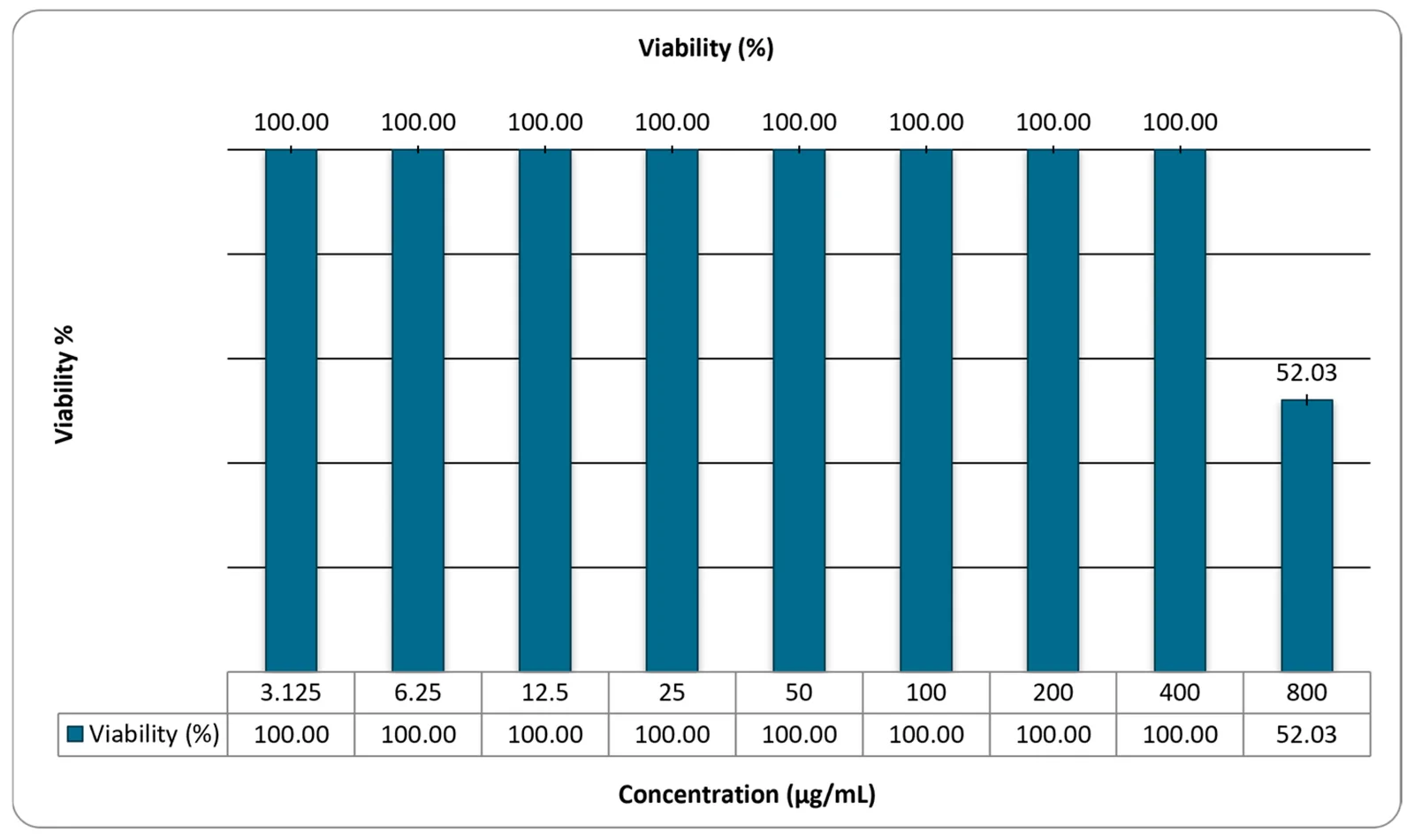
Viability of bMECs following treatment with different concentrations of 4-HCA, as determined by MTT assay. Values represent mean ± SEM.

**Scheme 2:**
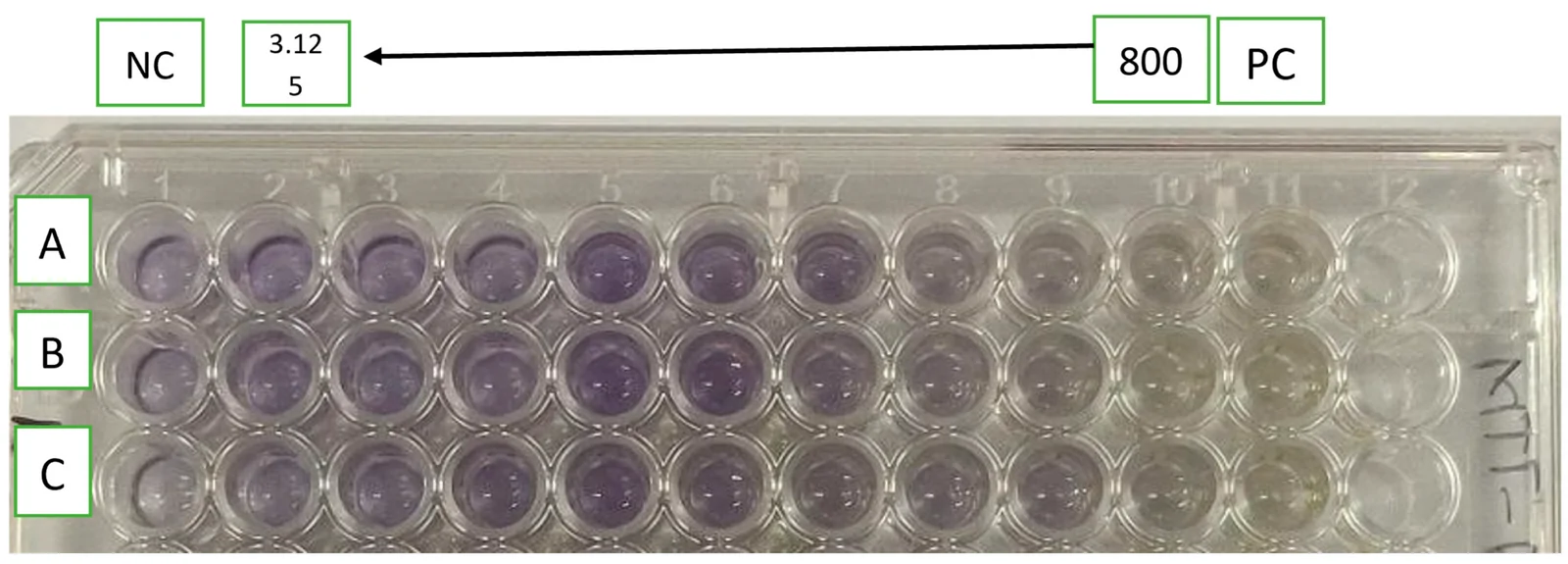
Showing end point of MTT assay, which is a purple colour read at the absorbance of 570 nm using a microplate reader. Rows A–C show three replicates of Trans-p-Coumaric acid starting with the concentration ranging from 800 µg/mL to 3.125 µg/mL; column 11—DMSO positive control; column 1—no treatment (control).

The effect of 4-HCA on bMEC viability was evaluated across concentrations ranging from 3.125 to 800 µg/mL. Cell viability remained at 100.00 ± 0.70% between 3.125 and 400 µg/mL. At the highest tested concentration of 800 µg/mL, viability decreased to 52.03 ± 1.08%. Importantly, the concentrations used for the qRT-PCR experiments, 50 and 100 µg/mL, showed 100.00 ± 0.70% viability under the experimental conditions (Scheme 2). These results indicate that no reduction in MTT-based metabolic viability was detected at the concentrations used for cytokine-expression analysis, whereas a marked reduction occurred at 800 µg/mL (Table 4 and Table 5 and Figure 9).

## 3. Discussion

Bovine mastitis remains a major challenge to dairy production due to its complex etiology, economic burden, and increasing prevalence of AMR. The present study evaluated the antimicrobial and immunomodulatory potential of 4-HCA, a plant-derived phenolic acid, against a diverse panel of mastitis-associated bacterial pathogens and in in vitro mammary epithelial inflammatory models. The findings collectively support the potential of 4-HCA as a therapeutic agent for the management of bovine mastitis.

### 3.1. Antimicrobial Activity of 4-HCA Against Mastitis-Associated Pathogens

Both agar well diffusion and disk diffusion assays revealed that 4-HCA displays broad-spectrum antibacterial efficacy against a diverse array of Gram-positive and Gram-negative bacterial strains recovered from cases of subclinical mastitis. Notably, *Staphylococcus aureus*, a major contagious mastitis pathogen known for its biofilm-forming ability and antibiotic tolerance, exhibited the highest susceptibility, with zones of inhibition reaching up to 12 mm at both tested concentrations. This is of particular relevance, as *S. aureus* infections are often refractory to conventional antibiotic therapy and contribute significantly to chronic mastitis.

Other pathogens frequently implicated in environmental and opportunistic mastitis, including *Enterococcus faecium*, *Pseudomonas putida*, *Shigella sonnei*, *Serratia liquefaciens*, and *Staphylococcus epidermidis*, also showed consistent inhibition. Partial or absent inhibition was observed in organisms such as *Raoultella planticola*, *Hafnia alvei*, and *Streptococcus agalactiae* highlights species-specific variability in susceptibility, which may be attributed to differences in outer membrane permeability, efflux pump activity, or intrinsic resistance mechanisms.

These findings are consistent with previous reports describing the antibacterial efficacy of plant-derived phenolic compounds against mastitis pathogens. Earlier studies are similar and reported selective sensitivity of Gram-positive bacteria to phytochemicals and essential oils, supporting the concept that phytochemicals can disrupt bacterial membranes and metabolic processes [20,21].

### 3.2. MIC and MBC Findings and Isolate-Dependent Activity

The MIC and MBC assays provided quantitative confirmation of the antimicrobial potency of 4-HCA. The average MIC of 0.625 mg/mL and MBC of 5 mg/mL across a broad range of bacterial isolates, including biofilm-forming strains. Importantly, 4-HCA demonstrated planktonic antimicrobial activity against isolates previously classified as strong, moderate, or weak biofilm producers. However, the present assays did not directly evaluate inhibition of biofilm formation or eradication of established biofilms.

The calculated MBC/MIC ratios ranged from 4 to 16. Six isolates showed a ratio of 4, six showed a ratio of 8, and two showed a ratio of 16. Using an MBC/MIC ratio of ≤4 as the operational bactericidal threshold, only six isolates met the criterion, whereas the remaining eight isolates did not. Therefore, the results do not support a generalized conclusion that 4-HCA was bactericidal against most of the evaluated isolates. These results align with other findings, which reported that p-coumaric acid increases bacterial membrane permeability, leading to irreversible damage to both the outer and cytoplasmic membranes [22,23]. Such a mechanism is particularly advantageous in combating biofilm-embedded bacteria, which are otherwise protected from antibiotic penetration.

### 3.3. Differential Immunomodulatory Effects on Bovine Mammary Epithelial Cells

Beyond direct antibacterial action, mastitis therapeutics must also mitigate the excessive inflammatory response that contributes to mammary tissue damage and reduced milk yield. In this context, the qRT-PCR analysis provided critical insights into the immunomodulatory role of 4-HCA in bMECs.

In the *S. aureus*-induced inflammatory model, 4-HCA treatment resulted in marked downregulation of TNF-α expression, a key pro-inflammatory cytokine involved in leukocyte recruitment, cytokine amplification, and tissue damage. The suppression of TNF-α suggests that 4-HCA may help limit excessive inflammatory signaling during mastitis. Interestingly, IL-1 and IL-6 expression showed upregulation in certain treatment groups. This phenomenon may reflect their dual role in host defense and tissue repair, as these cytokines are also involved in acute phase responses and epithelial regeneration rather than being exclusively pathogenic in mastitis, as supported by a previous study [19].

In the *E. coli*-infected bMEC model, 4-HCA reduced the expression of IL-1, IL-6, and TNF-α at both tested concentrations. IL-1 expression decreased to 0.33-fold at 100 μg/mL and 0.46-fold at 50 μg/mL. The reduction in TNF-α and IL-6 expression was also more pronounced at 100 μg/mL, indicating a concentration-dependent immunomodulatory response. These findings support an anti-inflammatory effect in the *E. coli* model; however, because the *S. aureus* model showed increased IL-1 and IL-6 expression, the overall activity of 4-HCA should be described as pathogen- and cytokine-dependent rather than uniformly anti-inflammatory.

### 3.4. Cell-Viability Findings and Study Limitations

Quantitative MTT analysis showed no reduction in bMEC metabolic viability at 4-HCA concentrations from 3.125 to 400 µg/mL under the evaluated experimental conditions. Thus, the concentrations used in the qRT-PCR experiments, 50 and 100 µg/mL, did not reduce MTT-based viability. In contrast, viability decreased to approximately 52% at 800 µg/mL, demonstrating reduced cellular tolerance at the highest tested concentration. These results support the cellular compatibility of the concentrations used for cytokine-expression analysis under the specific in vitro conditions of this study. However, MTT primarily measures metabolic activity and should not be interpreted as comprehensive evidence of cellular safety. Additional assays evaluating membrane integrity, apoptosis, oxidative stress, and longer exposure periods are required before preclinical safety or therapeutic claims can be made.

### 3.5. The Immunomodulatory Effects

The immunomodulatory effects observed in this study are comparable to those reported for other phenolic compounds such as chlorogenic acid and gallic acid. Gong et al. [24] demonstrated that chlorogenic acid reduced *S. aureus* invasiveness and downregulated IL-6 and TLR-2 expression in bMECs, while Mektrirat et al. [25] reported that gallic acid attenuated inflammation by modulating IL-1, IL-6, and TNF-α expression.

Importantly, phytochemicals like 4-HCA act through multiple mechanisms, including antimicrobial activity, inhibition of inflammatory signaling pathways (COX-2, iNOS, MAPKs), and immune cell modulation. Such multimodal action reduces the likelihood of resistance development, making them particularly attractive in the context of AMR and the One Health framework.

## 4. Materials and Methods

### 4.1. Bacterial Strains and Cultural Conditions

The bacteria used in the present study were isolated from milk samples that were California Mastitis Test (CMT) 2+ positive, collected from different locations in and around Jammu city, Jammu and Kashmir. The stock cultures were maintained in Mueller–Hinton broth at 4 °C. A sterile spreader (HiMedia Laboratories Pvt. Ltd., Mumbai. India) was used for inoculation of the organism across respective media. The following is the list of bovine mastitis-origin bacterial isolates used for this research work (Table 6).

### 4.2. Estimation of Biofilm Formation

#### 4.2.1. Tube Method

Overnight bacterial cultures were inoculated into 10 mL of Tryptone Soya HiCynth Broth (TSB) enriched with 1% glucose and incubated statically at 37 °C for 24 h, adhering to the protocol established by Christensen et al. [26]. Following incubation, the broth medium was decanted, and the assay tubes were inverted for 15 min to drain residual liquid. Adherent biofilms were stained using 0.1% (*w*/*v*) crystal violet solution (HiMedia Laboratories Pvt. Ltd., Mumbai. India) for 10 min, after which unabsorbed stain was removed by rinsing with sterile phosphate-buffered saline (PBS, pH 7.2). The tubes were subsequently air-dried in an inverted orientation and evaluated for biofilm development. The formation of a visible stained ring or film coating the internal walls and base of the tube was scored as a positive indicator of biofilm synthesis. All assays were performed in biological and technical triplicate, with biofilm-forming isolates categorized quantitatively into strong, moderate, weak, or non-producing phenotypes.

#### 4.2.2. Microtiter Plate Assay

Overnight bacterial cultures obtained from single pure colonies were inoculated into a 96-well plate containing TSB supplemented with 1% glucose. Each microtiter well was inoculated with an individual bacterial isolate and incubated at 37 °C for 24 h. Biofilm biomass was quantified spectrophotometrically using an Epoch microplate reader (Biotek Instruments, Winooski, VT, USA) by measuring the optical density (OD) at a wavelength of 600 nm. Data are presented as the arithmetic mean ± standard deviation (SD) and summarized in tabular format.

### 4.3. Antimicrobial Activity of Phytochemical

Phytochemical (4-HCA) was purchased from TCI Chemicals, Japan (Cat. C0393). A total 14 different bacteria such as *Hafina alvei*, *Klebsiella quasipneumoniae*, *Escherichia coli*, *Mammilococcus sciuri*, *Enterococcus faecalis*, *Streptococcus agalactiae*, *Shigella sonnei*, *Staphylococcus aureus*, *Staphylococcus epidermis*, *Enterococcus faecium*, *Pseudomonas putida*, *Serratia liquefaciens*, *Proteus mirabilis*, *and Raoultella planticola* were used against 4-HCA for determination of antimicrobial activity using different methods, like agar well diffusion method, disc diffusion method, minimum inhibitory concentration (MIC) and minimum bactericidal concentration (MBC).

#### 4.3.1. Agar Well Diffusion Method

The antimicrobial activity of 4-HCA was evaluated individually via the agar well diffusion assay, following the methodology described by Srinivasan et al. [27]. Sterile Mueller–Hinton agar (20 mL) was poured into Petri plates (HiMedia Laboratories Pvt. Ltd., Mumbai. India) under aseptic conditions. Upon medium solidification, the agar surfaces were uniformly inoculated by swabbing with standardized bacterial suspensions (10^8^ cells/mL). Wells measuring 5 mm in diameter were created in the agar using a sterile cork borer (3/16 inches). Subsequently, 50 µL of 4-HCA solution at concentrations of 20 mg/mL and 50 mg/mL was dispensed into the respective wells. The plates were maintained at room temperature for 1 h to allow pre-diffusion of the test compound, followed by incubation at 37 °C for 18 to 24 h. DMSO served as the negative control, while ciprofloxacin was utilized as the positive reference standard. Antibacterial efficacy was quantified by measuring the resulting diameter of the clear zone of inhibition in millimeters (mm). All assays were executed in biological and technical triplicate.

#### 4.3.2. Disc Diffusion Method

Bacterial activation was achieved by inoculating a loopful of culture into nutrient broth followed by a 6 h incubation period to adjust the cellular suspension to a 0.5 McFarland standard turbidity (10^8^ cells/mL). The standardized inoculum was then evenly streaked across Mueller–Hinton agar plates. Aliquots (40 µL) of 4-HCA at test concentrations of 20 mg/mL and 50 mg/mL were applied onto sterile filter paper discs (6 mm diameter) (HiMedia Laboratories Pvt. Ltd., Mumbai. India) and allowed to dry completely. The impregnated discs were subsequently placed onto the surface of the inoculated agar plates. DMSO and ciprofloxacin served as the negative and positive controls, respectively. Following incubation at 37 °C for 24 h, antimicrobial efficacy was evaluated by measuring the diameter of the resulting zones of inhibition. All assays were performed in triplicate in accordance with the established protocols [28,29].

#### 4.3.3. Minimum Inhibitory Concentration (MIC) and Minimum Bactericidal Concentration (MBC)

The MIC of 4-HCA was ascertained using the broth microdilution technique detailed by Agyare et al. [30]. Ninety-six-well microtiter plates (HiMedia Laboratories Pvt. Ltd., Mumbai. India) were prepared by dispensing 100 µL of Mueller–Hinton broth into each well via a multichannel pipette (HiMedia Laboratories Pvt. Ltd., Mumbai. India). Serial twofold dilutions of 4-HCA (100 µL), spanning a concentration gradient from 0.001 mg/mL to 10 mg/mL, were subsequently incorporated. The wells were then inoculated with 10 µL of standardized bacterial suspension (1.0 × 10^5^ CFU/mL) and incubated at 37 °C for 18 to 24 h. Absorbance readings were acquired using a microplate ELISA reader (Biotek Instruments, Vermont, USA). Ciprofloxacin, tested at concentrations between 0.002 and 2 mg/mL served as the reference antibiotic control. All assays were executed in independent triplicates to ensure data reliability. Following incubation, 10 µL aliquots from each well dilution were spot-inoculated onto Mueller–Hinton agar plates and incubated at 37 °C for 24 h. The MBC was established as the lowest test concentration yielding absolute absence of colonial growth.

### 4.4. Quantitative Real-Time Polymerase Chain Reaction (qRT-PCR)

To evaluate messenger mRNA expression profiles, bovine mammary epithelial cells (bMECs) were seeded into six-well culture plates at a density of 1 × 10^7^ cells per well and maintained at 37 °C in a humidified incubator containing 5% CO_2_. The cellular preparations were subsequently allocated into four distinct experimental cohorts:

(1) Untreated infected control group: basal growth medium (DMEM) with bMECs infected with *S. aureus* and *E. coli* separately @ 1 × 10^6^ CFU/mL for 6 h (n = 3);
(2) Ciprofloxacin-treated infected control group: bMECs infected with *S. aureus* (JNB1032) and *E. coli* (JNB1043) separately (1 × 10^6^ CFU/mL) treated with 100 μg/mL ciprofloxacin (n = 3);
(3) Treatment group: bMECs infected with *S. aureus* (JNB1032) and *E. coli* (JNB1043) separately (1 × 10^6^ CFU/mL) treated with 100 μg/mL 4-HCA for 12 h (n = 3); and
(4) Treatment group: infected bMECs infected with *S. aureus* (JNB1032) and *E. coli* (JNB1043) separately (1 × 10^6^ CFU/mL) treated with 50 μg/mL 4-HCA for 12 h (n = 3).

Total RNA was isolated from the cultured cells using the trizol method. A reverse transcription reaction mixture was prepared containing 0.3 μL forward and reverse primers, 2 μL cDNA, 7.4 μL nuclease free water, and 10 μL Go Taq qPCR master mix, making a final volume of 20 μL. cDNA was synthesized according to the instructions of the Promega GoScript^TM^ Reverse Transcription System kit. The qPCR amplification was performed using the pre-defined profile option in MyGo Mini software. The thermal cycling conditions were programmed as follows: an initial activation and denaturation step at 95 °C for 5 min (1 cycle), followed by 40 cycles of a three-step amplification profile consisting of denaturation at 95 °C for 20 s, primer-specific annealing at the optimized temperature for 15 s, and extension at 72 °C for 15 s. Following amplification, melt-curve analysis was executed, comprising an initial thermal equilibration at 95 °C for 5 s, a baseline stabilization step at 70 °C for 1 min, and a gradual linear temperature increase to 95 °C (held for 40 s). The thermal protocol concluded with a final cooling hold at 40 °C for 30 s [31].

Prior to experimental sample quantification, the amplification efficiency of each primer pair was validated using standard template dilution curves. Specific primer sequences are detailed in Table 7. Relative transcriptional expression levels of target genes were normalized against the housekeeping gene glyceraldehyde 3-phosphate dehydrogenase (GAPDH, NM_001034034) and evaluated using the comparative 2^−ΔΔCT^ method.

### 4.5. MTT Cell-Viability Assay

To conduct the assay, 5 × 10^4^ cells/mL were placed in a sterile 96-well microplate with a final volume of 100 μL of complete Dulbecco’s Modified Eagle Medium (DMEM). The plate was then incubated at 37 °C for 24 h, with humidity and 5% CO_2_ in a CO_2_ incubator (Galaxy 170S, New Brunswick, Eppendorf, Hamburg, Germany) to facilitate the formation of a confluent monolayer. The cells were then subjected to various doses of 4-HCA (3.125- 800 μg/mL) following the methodology described by [32] with some modification and incubated for 24 h at 37 °C in an atmosphere wet with 5% CO_2_. Cells were exposed to nine concentrations of 4-HCA prepared by two-fold serial dilution: 3.125, 6.25, 12.5, 25, 50, 100, 200, 400, and 800 µg/mL. Untreated cells served as the viability control. Each concentration was evaluated in triplicate. Bovine Mammary Epithelial Cells were used as a control with a complete DMEM alone. After this, the culture medium was discarded, and the cell viability was evaluated by the colorimetric method of reduction of MTT (3-(4,5-dimethylthiazol-2-yl)-2,5-diphenyltetrazole bromide) as described by [33]. Then, 90 μL of DMEM medium and 10 μL of the MTT solution were added. The plate was incubated at 37 °C with 5% CO_2_ for four hrs. Finally, 100 μL of DMSO per well was added to dissolve the formazan crystals that resulted from the MTT conversion. Results were determined by reading OD570 nm using an ELISA plate reader (Biotek Instruments, Winooski, VT, USA).

Cell viability (%) = (OD of treated cells/mean OD of untreated cells) × 100.

Results are presented as mean viability percentage ± SEM.

### 4.6. Statistical Analysis

The qRT-PCR results are presented as mean relative expression ± SEM and were analysed using GraphPad Prism version 10.0.0. Statistical analysis was performed using the individual replicate ΔCt values, whereas relative expression was presented as 2^−ΔΔCT^ fold change. For each bacterial infection model, differences among the untreated infected control, ciprofloxacin-treated infected control, 100 µg/mL 4-HCA treatment, and 50 µg/mL 4-HCA treatment were evaluated using two-way analysis of variance followed by Tukey’s multiple-comparison test. Differences were considered statistically significant at *p* < 0.05.

## 5. Conclusions

The findings of this investigation indicate that the naturally occurring phenolic phytochemical 4-HCA possesses antimicrobial and immunomodulatory properties against primary etiological agents of bovine mastitis, notably *Staphylococcus aureus* and *Escherichia coli*. The consistent inhibition zones against biofilm and non-biofilm formers highlights its therapeutic potential. Additionally, 4-HCA significantly modulated pro-inflammatory cytokines (TNF-α, IL-1, and IL-6) in bovine mammary epithelial cells. These findings support 4-HCA as a promising alternative or adjunct therapy for mastitis management in the era of antimicrobial resistance.

## Figures and Tables

**Table 1:** MIC and MBC of 4-HCA against various bovine mastitis-origin bacterial isolates.

Bacterial Isolates	MIC (mg/mL) of 4-HCA	MBC (mg/mL) of 4-HCA	MBC/MIC
*H. alvei*	0.625	5	8
*K. quasipneumoniae*	0.625	2.5	4
*E. coli*	0.625	5	8
*M. sciuri*	0.625	5	8
*E faecalis*	1.25	5	4
*S. agalactiae*	0.625	5	8
*S. sonnei*	1.25	5	4
*S. aureus*	0.625	5	8
*S. epidermis*	0.625	10	16
*E. faecium*	1.25	5	4
*P. putida*	1.25	10	8
*S. liquefaciens*	1.25	5	4
*P. mirabilis*	1.25	5	4
*R. planticola*	0.625	10	16

**Table 2:** Relative gene expression ratio (Mean ± SEM) of bMECs infected with *S. aureus*.

Gene	Negative Control	Positive Control (Standard)	Treatment (100 μg/mL)	Treatment (50 μg/mL)
IL-1	1 ^B^	0.84 ± 0.01 ^A^	1.42 ± 0.01 ^C^	4.35 ± 0.02 ^D^
IL-6	1 ^A^	10.1 ± 0.1 ^C^	4.6 ± 0 ^B^	12.2 ± 0.1 ^D^
TNF-α	1 ^D^	0.03 ± 0 ^A^	0.08 ± 0 ^C^	0.04 ± 0 ^B^

Means with different superscripts within the row differ from each other (*p* < 0.05).

**Table 3:** Relative gene expression ratio (in folds) of bMECs infected with *E. coli*.

Gene	Negative Control	Positive Control (Standard)	Treatment (100 μg/mL)	Treatment (50 μg/mL)
IL-1	1 ^D^	0.31 ± 0.02 ^A^	0.33 ± 0.01 ^B^	0.46 ± 0.02 ^C^
IL-6	1 ^D^	0.13 ± 0.01 ^A^	0.75 ± 0.04 ^B^	0.96 ± 0.01 ^C^
TNF-α	1 ^D^	0.08 ^A^	0.06 ± 0.01 ^B^	0.20 ± 0.01 ^C^

Mean with different superscripts within the row differ from each other (*p* < 0.05).

**Table 4:** MTT microplate absorbance readings for bMECs treated with 4-HCA.

Concentration (µg/mL)	Replicate 1 OD	Replicate 2 OD	Replicate 3 OD
3.125	0.82	0.83	0.81
6.25	0.81	0.82	0.83
12.5	0.82	0.81	0.83
25	0.83	0.82	0.81
50	0.81	0.83	0.82
100	0.82	0.81	0.83
200	0.83	0.82	0.81
400	0.82	0.83	0.81
800	0.44	0.41	0.43

**Table 5:** Effect of 4-HCA concentration on bMEC viability determined by MTT assay.

Concentration (µg/mL)	Mean OD	Viability (%)	SEM
3.125	0.820	100.00	0.70
6.25	0.820	100.00	0.70
12.5	0.820	100.00	0.70
25	0.820	100.00	0.70
50	0.820	100.00	0.70
100	0.820	100.00	0.70
200	0.820	100.00	0.70
400	0.820	100.00	0.70
800	0.427	52.03	1.08

**Table 6:** Bacteria isolated from bovine mastitis milk.

S. No.	Isolates	Isolate Number
1.	*E. faecium*	BM1210
2.	*S. aureus*	BM1227
3.	*R. planticola*	BM1207
4.	*P. putida*	BM1203
5.	*S. agalactiae*	BM1222
6.	*E. coli*	BM1221
7.	*P. mirabilis*	BM1208
8.	*M. sciuri*	BM1214
9.	*H. alvei*	BM1206
10.	*E. faecalis*	BM1212
11.	*S. sonnei*	BM1213
12.	*S. liquefaciens*	BM1204
13.	*S. epidermis*	BM1223
14.	*K. quasipneumoniae*	BM1205

**Table 7:** List of primers.

S.No.	Gene	Sequence	Annealing Temp
1.	IL-1	F- CTTGGGAGGACTGAGGCTAC	57.4 °C
		R- CGGCTTATTCCAACTGCTGT	
2.	IL-6	F- CGAAAGAGAGCTCCATCTGCC	54 °C
		R- GCGCTTAATGAGAGCTTCGG	
3.	TNF-α	F- GCTGACGGGCTTTACCTCAT	58.9 °C
		R- TGGTAGGAGACTGCAATGCG	

## Data Availability

The datasets used and analyzed during the current study are available from the corresponding authors upon reasonable request.
